# Association of overweight and obesity with cardiovascular risk factors in patients with atherosclerotic diseases

**DOI:** 10.2478/jomb-2019-0027

**Published:** 2020-01-23

**Authors:** Milos Maksimovic, Hristina Vlajinac, Djordje Radak, Jelena Marinkovic, Jadranka Maksimovic, Jagoda Jorga

**Affiliations:** 1 University of Belgrade, Faculty of Medicine, Institute of Hygiene and Medical Ecology, Belgrade; 2 University of Belgrade, Faculty of Medicine, Institute of Epidemiology, Belgrade; 3 University of Belgrade, Faculty of Medicine, Dedinje Cardiovascular Institute, Department of Vascular Surgery, Belgrade; 4 University of Belgrade, Faculty of Medicine, Institute of Medical Statistics and Informatics, Belgrade

**Keywords:** obesity, atherosclerosis, risk factors, carotid disease, peripheral arterial disease, periferna arterijska bolest, karotidna bolest, faktori rizika, ateroskleroza, gojaznost

## Abstract

**Background:**

The aim of this study was to compare demographic, clinical and biochemical characteristics, including inflammatory markers, according to the nutritional status of patients with verified atherosclerotic disease.

**Methods:**

This cross-sectional study involved 1045 consecutive patients with verified carotid disease or peripheral arterial disease (PAD). Anthropometric parameters and data on cardiovascular risk factors and therapy for hypertension and hyperlipidemia were collected for all participants.

**Results:**

Carotid disease was positively and PAD was negatively associated with body mass index (BMI). Negative association between obesity and PAD was significant only in former smokers, not in current smokers or in patients who never smoked. Overweight and general obesity were significantly related to metabolic syndrome (p < 0.001), lower values of high - density lipoprotein cholesterol (p < 0.001), increased triglycerides (p < 0.001), hyperglycemia (p < 0.001), self-reported diabetes (p < 0.001), hypertension (p < 0.001), high serum uric acid (p < 0.001), increased high sensitivity C-reactive protein (p = 0.020) and former smoking (p = 0.005) after adjustment for age, gender and type of disease. Antihypertensive therapy seems to be less effective in patients who are overweight and obese.

**Conclusions:**

In conclusion, overweight and general obesity were significantly related to several cardiovascular risk factors.

## Abstract (sr)

**Uvod:**

Cilj ove studije je bio da se porede demografske, kliničke i biohemijske karakteristike, uključujući inflamatore markere, sa stanjem uhranjenosti kod pacijenata obolelih od aterosklerotske bolesti.

**Metode:**

Ova studija preseka je obuhvatila 1045 pacijenata sa dijagnostikovanom karotidnom i perifernom arterijskom bolešću. Antropometrijski parametri i podaci o faktorima rizika za kardiovaskularne bolesti i terapiju za hipertenziju i hiperlipidemiju su prikupljeni od svih pacijenata.

**Rezultati:**

Karotidna bolest je bila povezana, dok periferna arterijska bolest nije bila povezana sa indeksom telesne mase (ITM). Negativna veza između gojaznosti i periferne arterijske bolesti je zabeležena samo kod bivših pušača, a ne i kod sadašnjih i kod onih koji nisu nikada pušili. Predgojaznost i gojaznost su bile značajno povezane sa metaboličkim sindromom (p < 0,001), smanjenim nivoom HDL holesterola (p < 0,001), povišenim nivoom triglicerida (p < 0,001), hiperglikemijom (p < 0,001), šećernom bolešću (p < 0,001), hipertenzijom (p < 0,001), povišenim nivoom mokraćne kiseline (p < 0,001), povišenim nivoom ultra senzitivnog C-reaktivnog proteina (p = 0,020) bivšim pušačima (p = 0,005) posle prilagođavanja na godine, pol i vrstu bolesti. Antihipertenzivna terapija izgleda da ima sma njen efekat kod pacijenata sa prekomernom telesnom masom i gojaznošću.

**Zaključak:**

Prekomerna telesna masa i gojaznost opšteg tipa su bile značajno povezane sa nekoliko faktora rizika za kardiovaskularne bolesti.

## Introduction

Obesity has become a public health problem in many countries over the past decades [1]. Data from the National Health Survey in 2013 show that 35.1% of the adult population in Serbia (≥20 years old) are overweight (body mass index -BMI 25.0-29.9 kg/m^2^) and 21.2% obese (BMI ≥ 30 kg/m^2^) [2]. In children 7-19 years old an increase in the prevalence of obesity was observed between 2000 and 2006 (overweight from 8.2 to 11.6% and obese from 4.4 to 6.4%).

Data have been accumulating showing that obesity is associated with significant morbidity and mortality, especially with cardiovascular disease (CVD), being an independent risk factor for CVD [3]. Even obesity in childhood and adolescence is associated with increased risk for cardiovascular diseases later in life [4]. Obesity is also related to many cardiovascular risk factors which increase the risk for CVD [5].

In a study including patients with established coronary heart disease, De Bacquer et al. [6] found that overweight and obese coronary patients are at particularly high risk for further cardiovascular complications due to increased number of risk factors levels and their insufficient therapeutic control.

The aim of the present study was to compare demographic, clinical and biochemical characteristics, including inflammatory markers, according to the nutritional status of patients with verified atherosclerotic disease (carotid and peripheral).

## Materials and Methods

This cross-sectional study involved 1045 consecutive patients who were referred to the Vascular Surgery Clinic Dedinje in Belgrade, Serbia, because of carotid disease or peripheral arterial disease (PAD). The study was conducted during the period April 2006 - November 2007.

The study included subjects who had symptoms of cerebral ischemia (amaurosis fugax, transient ischemic attack or stroke) and carotid stenosis ≥50%, according to the North American Symptomatic Carotid Endarterectomy Trial (NASCET) criteria [7], and patients with symptomatic peripheral arterial disease (PAD) (claudication, rest pain or gangrene). The high resolution B-mode ultrasonography HDI, ATL (»Advanced Technology Laboratories«, »Cineloop«) 3500 (Philips Ultrasound, Eindhoven, The Netherlands) was used for estimation of carotid disease. PAD was defined as an ankle-brachial index (ABI) of <0.9 according to Doppler sonography. ABI is highly sensitive (90%) and highly specific (95%) for PAD [8]. Doppler sonography was applied on both lower limbs. As the ABI was recorded the lowest value.

In the study were not included patients under 18 years of age, and patients with malignant disease, previous endarterectomy, and rheumatoid arthritis.

Using questionnaire from all participants were collected data on demographic characteristics (age, sex, education), anthropometric and lifestyle characteristic (body height, body weight, waist circumference, smoking, alcohol consumption, and physical activity, as well as data on some diseases in personal history (diabetes, ischemic heart disease, aneurysm and other atherosclerotic diseases), and data on treatment for hyper tension and hyperlipidemia.

Body Mass Index (BMI), calculated as weight (kg) divided by height (m^2^) was used for assessment of body composition. Body weight was categorized as: normal weight (BMI <25.0 kg/m^2^), overweight (BMI 25.0-29.9 kg/m^2^) and obesity (BMI ≥30.0 kg/m^2^) [9]. Blood pressure (BP) was measured according to the recommendations in the Seventh Report of the Joint National Committee on Prevention, Detection, Evaluation, and Treatment of High Blood Pressure [10]. Blood samples were obtained after an overnight fast and abstention from liquids (except for water) in order to estimate levels of fasting plasma glucose (FPG), total cholesterol (TC), low-density lipoprotein cholesterol (HDL-C), high-density lipoprotein cholesterol (HDL-C), triglycerides (TG), serum uric acid (SUA), high sensitivity C-reactive protein (hsCRP) and fibrinogen. The high value of hsCRP was assessed according to the Centers for Disease Control (CDC) recommendation as ≥3 mg/L [11]. The National Cho le sterol Education Program III (NCEP) criteria [12] were used for definition of metabolic syndrome.

According to smoking, each subject was classified as a non-smoker, former smoker or current smoker. The number of cigarettes smoked and duration of smoking were expressed as pack years. Alcohol consumption was analyzed as a) alcohol consumption, yes (current and former drinking) vs. never, and b) by calculating the total dose of alcohol consumption for each participant by adding all the individual beverages (50 mL of brandy/hard liquor/beer, and 200 mL of wine) weighted to their alcohol content (30% in brandy, 40% in hard liquor, 12% in wine and 3.5% in beer).

Physical activity was assessed on the basis of data for the previous month. It was defined as any type of non-occupational physical exercise lasting more than 30 min per day. As a physically active were considered only those who exercised more than once per week.

### Statistical analysis

Chi square test, t-test and one-way ANOVA were used for the analyses of differences between BMI categories in distribution of patients by age, sex, atherosclerotic disease category, pack years of smoking and quantity of alcohol consumption. The association between categories of BMI and these variables was analyzed by contingency coefficient. Multivariate logistic regression analysis model was used with three aims. Firstly – to analyze the relationship between BMI and PAD according to smoking status with adjustment for age and sex. Secondly – to analyze categories of BMI (lowest category is a referent one) as a potential risk factors of selected outcome variables (different cardiovascular risk factors). Association of BMI with different cardiovascular risk factors was assessed after adjustment for age, sex and type of atherosclerotic disease (carotid or PAD). Thirdly – to analyze and eventually establish the relationship between BMI and high BP, dyslipidemia and elevated hsCRP in treated (antihypertensive and lipid lowering drug intake) and untreated patients. The adjustments were made for age, sex, type of atherosclerotic disease, diabetes, education, current smoking and former smoking.

A level of α = 0.05 was used to indicate statistical significance. Data were analyzed using Statistical Package for the Social Sciences, version 20 (SPSS Inc., Chicago, IL, USA).

### Ethical approval

The study was given ethical approval by the Ethics Committee at the School of Medicine in Belgrade. All patients gave their written, informed consent.

## Results

Data were collected for 1045 patients, 657 with carotid disease and 388 with PAD. Out of them 361 patients (34.55%) had normal weight, 487 patients (46.6%) were overweight, and 197 (18.85%) were obese. Normal, overweight and obese patients significantly differ in age, gender and type of atherosclerotic disease (carotid or peripheral), but not in the severity of the disease (Table 1). Obese patients were the most frequent among subjects 55-64 years old and more frequent among women than men, and they had more frequently carotid disease and less frequently PAD. Difference in type of atherosclerotic disease was independent of age and gender differences.

**Table 1:** Age, sex and atherosclerotic disease category of patients according to body mass index ^a^ According to chi-square test

Variable	Body mass index			
	<25.0(n = 361)	25.0 – 29.9(n = 487)	≥ 30.0(n = 197)	
	No. (%)	No. (%)	No. (%)	*P* value^b^
Age: <55 55 – 64 ≥65	64 (17.73)103 (28.53)194 (53.74)	67 (13.76)174 (35.73)246 (50.51)	21 (10.66)83 (42.13)93 (47.21)	0.011
Sex: women men	107 (29.64)254 (70.36)	140 (28.75)347 (71.25)	82 (41.62)115 (58.36)	0.003
Carotid disease: Stroke Other	207 (57.34)123 (59.42)84 (40.58)	309 (63.45)189 (61.16)120 (38.83)	141 (71.57)85 (60.28)56 (39.72)	0.004 0.923
Peripheral arterial disease: Claudication Rest pain Gangrene	154 (42.66) 94 (61.04)12 (7.79)48 (31.17)	178 (36.55) 123 (69.10)17 (9.55)38 (21.35)	56 (28.43) 36 (64.28)3 (5.36)17 (30.36)	0.004 0.271

The analysis of the relationship between BMI and PAD by smoking status (Table 2) showed that frequency of PAD was lower among persons with increased BMI in all smoking subgroups. However, negative association between obesity and PAD was significant only in former smokers, not in current smokers or in patients who never smoked.

**Table 2:** Association between Peripheral Artery Disease (PAD) and Body Mass Index (BMI) by smoking status * Odds ratio and P value according to multivariate logistic regression analysis, adjusted on age and sex

	PAD in never smokers			PAD in former smokers			PAD in current Smokers		
BMI (kg/m^2^)	No/ Total	%	OR (P)*	No/Total	%	OR (P)*	No/Total	%	OR (P)*
< 25.0	12/72	16.7	1 (reference)	44/110	40.0	1 (reference)	98/179	54.7	1 (reference)
25.0 –29.9	17/129	13.2	0.77 (0.523	59/165	35.8	0.76 (0.29)	102/193	52.8	0.90 (0.633)
≥ 30	9/62	14.5	0.86 (0.761)	20/79	25.3	0.33 (0.015)	27/56	48.2	0.86 (0.630)

BMI was correlated with metabolic syndrome, lower values of HDL-C, abdominal obesity, increased triglycerides, hyperglycemia, diabetes, hypertension, high serum uric acid, increased hsCRP and former smoking, as well as with lipid lowering drug intake and antihypertensive therapy (Table 3). Overweight and general obesity, defined according to BMI, were significantly correlated with abdominal obesity - correlation coefficient was 0.534, *p* < 0.001. Significant inverse association was found with current smoking and significant positive association was found with former smoking. There was no significant association with education, total cholesterol, LDL-C, fibrinogen, and other atherosclerotic diseases, alcohol consumption and physical inactivity. Compared BMI groups did not significantly differ either in pack/year of smoking (*p* = 0.175) or daily alcohol consumption (*p* = 0.802).

**Table 3:** Prevalence of cardiovascular risk factors in patients according to body mass index; results of multivariate logistic regression analysis Notes: DBP, diastolic blood pressure; HDL-C, high-density lipoprotein cholesterol; hsCRP, high-sensitivity C-reactive protein; LDLC, low-density lipoprotein cholesterol; SBP, systolic blood pressure; SUA, serum uric acid. ^a^ Significance of differences between groups after adjustment for age, sex and type of atherosclerotic disease (carotid or peri pheral) ^b^ > 341 μmol/L (median value). ^c^ Fibrinogen was not measured in 12 patients (2 with normal weight, 9 with overweight and 1 obese patients). ^d^ Non-occupational physical exercise 0–4 times per month.

Variable		Body mass index		
	< 25.0 (n = 361)	25.0 – 29.9(n = 487)	≥ 30.0(n = 197)	
	n (%)	n (%)	n (%)	*P* value^a^
Metabolic syndrome	113 (31.30)	310 (63.65)	174 (88.32)	<0.001
Waist circumference				
≥ 88 cm in women and ≥102 cm in men	30 (8.3)	262 (53.8)	189 (95.9)	<0.001
Total cholesterol ≥ 5.20 mmol/L	177 (49.03)	245 (50.31)	97 (49.23)	0.954
HDL-C ≤ 1.59 mmol/L	344 (95.29)	475 (97.54)	191 (96.95)	0.142
HDL-C 1.00 mmol/L	161 (44.60)	269 (55.24)	111 (56.34)	<0.001
LDL-C ≥ 4.10 mmol/L	93 (25.76)	119 (24.43)	44 (22.33)	0.340
Triglycerides ≥ 1.70 mmol/L	134 (37.12)	264 (54.21)	117 (59.39)	<0.001
Triglycerides ≥ 2.30 mmol/L	64 (17.73)	146 (29.98)	69 (35.02)	<0.001
Lipid lowering drug intake	130 (36.01)	205 (42.09)	100 (50.76)	0.002
Self-reported diabetes	114 (31.58)	178 (36.55)	93 (47.21)	<0.001
Fasting glucose ≥6.1 mmol/L	74 (20.50)	129 (26.49)	76 (38.58)	<0.001
SBP ≥ 140 mmHg				
and/or DBP ≥ 90 mmHg	204 (56.51)	336 (68.99)	145 (73.60)	<0.001
Antihypertensive therapy	299 (82.82)	436 (89.53)	181 (91.88)	0.002
High SUA_^b^_, μmol/L	145 (40.17)	255 (52.36)	119 (60.41)	<0.001
hsCRP ≥ 3 mg/L	140 (38.78)	209 (42.92)	92 (46.70)	0.020
Fibrinogen ≥ 4 g/L^c^	100 (27.85)	124 (25.94)	56 (28.57)	0.834
Other atherosclerotic disease in personal history:				
Ischemic heart disease	107 (29.6)	191 (39.2)	64 (32.5)	0.239
Carotid disease/peripheral artery disease	66 (18.3)	78 (16.0)	24 (12.2)	0.076
Anerysm	18 (5.0)	16 (3.3)	5 (2.5)	0.196
Years of education < 12	100 (27.70)	169 (34.70)	71 (36.04)	0.097
Current smoking	179 (49.58)	193 (39.63)	56 (28.43)	<0.001
Former smoking	110 (30.47)	165 (33.88)	79 (40.10)	0.005
Alcohol consumption (current and former drinking)	155 (42.94)	211 (43.33)	69 (35.03)	0.823
Physical inactivityd	332 (91.97)	437 (89.73)	183 (92.89)	0.867

According to data presented in Table 4, antihypertensive therapy seems to be less effective in patients who are overweight and obese. Lipid lowering drug intake (all patients, but 4 of them, used statins) showed favorable effect on total cholesterol and LDL-C levels, but not on HDL-C level. The effect on triglycerides level was present only in patients with normal weight). The favorable effect of lipid lowering drugs was also found for hsCRP. Among untreated patients the prevalence of hsCRP ≥3.0 mg/L increased significantly with the increase of BMI. In treated patients the prevalence of increased hsCRP was lower, particularly in obese patients.

**Table 4:** Prevalence of high blood pressure, dyslipidemia and elevated hsCRP in treated (antihypertensive and lipid lowering drug intake) and untreated patients according to body mass index – results of multivariate logistic regression analysis Notes: DBP, diastolic blood pressure; HDL-C, high-density lipoprotein cholesterol; hsCRP, high-sensitivity C-reactive protein; LDLC, low-density lipoprotein cholesterol; SBP, systolic blood pressure. ^a^ Significance of differences between groups after adjustment for age, sex, type of atherosclerotic disease (carotid or peripheral),diabetes, education, current smoking and former smoking ^b^ Not treated with BP lowering drugs ^c^ Treated with BP lowering drugs ^d^ Not treated with lipid lowering drugs ^e^ Treated with lipid lowering drugs

Variable	Body mass index			
	< 25.0 (n = 361)	25.0 – 29.9 (n = 487)	≥ 30.0 (n = 197)	
	No. (%)	No. (%)	No. (%)	P value^a^
SBP ≥140 mmHg and/or DBP ≥ 90 mmHg – NT^b^	20 (32.26)	29 (56.86)	11 (68.75)	0.008
SBP ≥140 mmHg and/or DBP ≥ 90 mmHg – T^c^	184 (61.54)	307 (70.41)	134 (74.03)	0.002
Triglycerides ≥ 1.70 mmol/L – NT^d^	96 (41.58)	153 (54.25)	54 (55.67)	0.003
Triglycerides ≥ 1.70 mmol/L – T^e^	38 (29.23)	111 (54.15)	63 (63.00)	<0.001
Total cholesterol ≥ 5.20 mmol/L – NTd	134 (58.01)	161 (57.09)	60 (61.86)	0.601
Total cholesterol ≥ 5.20 mmol/L – T^e^	43 (33.08)	84 (40.98)	37 (37.00)	0.597
LDL-C ≥ 3.40 mmol/L – NT^d^	128 (55.41)	157 (55.67)	54 (55.67)	0.746
LDL-C ≥ 3.40 mmol/L – T e	42 (32.31)	79 (38.54)	29 (29.00)	0.617
HDL-C 1.59 mmol/L – NT^d^	220 (95.24)	275 (97.52)	94 (96.91)	0.423
HDL-C 1.59 mmol/L – T^fe^	124 (95.38)	200 (97.56)	97 (97.00)	0.516
hsCRP ≥ 3.00 mg/L – NT^d^	90 (38.96)	123 (43.62)	59 (60.82)	<0.001
hsCRP ≥ 3.00 mg/L – T^e^	50 (38.46)	86 (41.95)	33 (33.00)	0.819

## Discussion

In the present study carotid disease was positively and PAD was negatively associated with BMI. Overweight and general obesity were significantly related to several cardiovascular risk factors.

There are numerous studies reporting the adverse effects of obesity, especially on cardiovascular health [5] [13]. Collaborative analyses of 57 prospective studies [13] showed that the progressive excess mortality above a BMI of 25.0 kg/m^2^ was mainly due to vascular disease. Obesity is a risk factor for coronary heart disease and carotid disease. However, data on the association of PAD and BMI are not consistent. In cross-sectional studies this association was not found, in some studies it was positive and in some of them it was negative [14] [15] [16] as it was in the present study. PAD is most common in older men in whom BMI may not be a proper indicator of obesity. In older men the loss of lean body mass can be substantial so that in spite of increased adiposity BMI may remain stable or may decrease [17].

Obesity seems to be associated with CVD in two ways, as an independent risk factor and through its association with other cardiovascular risk factors.

According to a large prospective study [18] obesity in middle age is an independent risk factor for CVD. People who were overweight and particularly those who were obese earlier in life had significantly higher risk of hospitalization and mortality from CVD in older age compared with those of normal weight with similar other cardiovascular risk factors at baseline.

Obesity is related to many cardiovascular risk factors such as glucose intolerance, metabolic syndrome, type 2 diabetes, hypertension and dyslipidemia [5] [19] and increasing evidence indicates that risk factors tend to cluster in obese individuals and may act synergistically to increase their risk for CVD [5] [20].

In the present study overweight and abdominal obesity were significantly related to metabolic syndrome and all its components. The inverse association of BMI with current smoking and positive association with former smoking is probably the result of weight increase frequently seen after cessation of smoking [21]. A possible explanation for different type of association (positive vs. negative) of BMI with carotid and PAD could be the difference in smoking status. Patients with PAD were more frequently current smokers than patients with carotid disease (58.5 vs. 30.9%, *p* < 0.001). There were no differences in the number of former smokers (31.7 vs. 35.2%, p = 0.283). It has been reported that current smokers have lower BMI compared with nonsmokers and former smokers [21]. Clair et al. [22] found that the current smoker had lower mean waist circumference, body fat percentage, and BMI compared with nonsmokers. In the study of Ix et al. [23] no associations of BMI with PAD prevalence or incidence were found when all participants were evaluated together. However, among persons who had never smoked and had good health status (self-assessed), a positive association of BMI with PAD prevalence and incidence was observed. In the present study a negative association between BMI and PAD was found in all subgroups classified by smoking status, but this relationship was significant only in former smokers. However, we had no data about health status of our participants and we did not ask them about the reasons for smoking cessation. It is possible that the presence of some other disease was associated with weight loss. It is also possible that association between BMI and PAD in persons who never smoked would be different if we had included in the analysis only those in good health.

Association of BMI and abdominal weight with serum uric acid found in the present study was reported by several investigations [24]. Elevated uric acid has been shown to be, at least in some studies, an independent predictor of coronary heart disease [25], stroke [26] and PAD [27] especially in women [28]. There is no agreement about the mechanism through which hyperuricemia increases the risk for CVD. There are suggestions that association between uric acid and carotid plaque may be attributable to metabolic syndrome, but also to some independent mechanisms [29].

In the present study increased hsCRP was present in 42% of all participants and it was significantly related to BMI. It is now recognized that adipose tissue is not only depot of fat, but an endocrine organ and that it is probably an important link between increased fat mass and insulin resistance. It also produces several inflammatory products that lead to atherogenesis [30]. CRP is related to various type of atherosclerosis [31]. However, it is uncertain whether CRP is a marker of CVD risk or if it is causally related to cardiovascular disease [31]. According to epidemiological studies CRP is an independent risk factor for coronary heart disease [32], stroke [33] and PAD [34], and high hsCRP is associated with severe PAD [35]. In contrast, Mendelian randomization tests of causality [31] suggest no causal association of CRP with coronary heart disease. Further studies are needed for understanding possible role of CRP in atherosclerosis.

Although in many investigations fibrinogen has been found to be associated with obesity especially in women [36] [37], in the present study level of fibrinogen did not significantly differ between BMI groups. Taking into account that fibrinogen has also been related to smoking [36] [38] [39] [40] this finding in our study might be explained by significantly higher percentage of current smokers among patients with normal BMI compared with overweight and obese patients.

The fact that 34% of patients in the present study were not overweight points to the importance of some additional factors, such as genetics, in the development of CVD.

In the present study in a considerable number of patients with carotid disease and PAD, cardiovascular risk factors persisted in spite of treatment and the response to antihypertensive drug therapy was worse in overweight patients. These findings are similar to the results of EUROASPIRE II study [6], performed on patients with coronary heart disease. In this study, in patients who had been using BP lowering agents, 56% of obese and 51% of overweight still had raised BP compared with 42% of normal weight patients, and this difference was significant. A similar result was obtained for the control of total cholesterol. Because of insufficient therapeutic control of raised BP and elevated total cholesterol, the authors of the EUROASPIRE II study [6] emphasize the importance of lifestyle modification in addition to more intensified drug treatment in overweight and obese coronary disease patients. We have no data on how long our patients have been treated and with what antihypertensive drugs, and we could not expect valid answer on possible question about regularity of taking prescribed BP lowering agents. It is probable that these data might help explain the results obtained.

The results of the present study confirm the efficacy of statin therapy on CRP and LDL-C reduction [41] and its limited effects on triglycerides and HDL-C [42].

The advantages of the present study are: the number of patients; the analysis of the negative association between BMI and PAD in subgroups according to smoking status as well as the analysis of the therapeutic control of manageable cardiovascular risk factors in relation to BMI. These analyses were performed in only few other studies or in patients with coronary heart disease. However, more detailed investigations, preferably prospective, are required in order to explain the findings of the study.

Our study has limitations. The cross-sectional design makes it difficult to judge causal relations. Another limitation is that study participants were from a single hospital and they did not represent all patients with carotid disease and PAD. In addition, we did not verify smoking status by CO monitoring or a biochemical method.

In conclusion, according to the results of the present study, elevated BMI is significantly related to several cardiovascular risk factors in patients with carotid disease and PAD.

*Funding sources*: This work was supported by Ministry of Education, Science and Technological Development, Republic of Serbia, through contract no. III41002.

### Conflict of interest statement

The authors declare that they have no conflict of interest.
